# First-Principles Study of Au-Doped InN Monolayer as Adsorbent and Gas Sensing Material for SF_6_ Decomposed Species

**DOI:** 10.3390/nano11071708

**Published:** 2021-06-29

**Authors:** Ruochen Peng, Qu Zhou, Wen Zeng

**Affiliations:** 1 College of Engineering and Technology, Southwest University, Chongqing 400715, China; ruochenpeng1207@163.com; 2 College of Materials Science and Engineering, Chongqing University, Chongqing 400044, China

**Keywords:** Au-InN, DFT, SF_6_ decomposed species, adsorption properties

## Abstract

As an insulating medium, sulfur hexafluoride (SF_6_) is extensively applied to electrical insulation equipment to ensure its normal operation. However, both partial discharge and overheating may cause SF_6_ to decompose, and then the insulation strength of electrical equipment will be reduced. The adsorption properties and sensing mechanisms of four SF_6_ decomposed components (HF, SO_2_, SOF_2_ and SO_2_F_2_) upon an Au-modified InN (Au-InN) monolayer were studied in this work based on first-principles theory. Meanwhile, the adsorption energy (E_ad_), charge transfer (Q_T_), deformation charge density (DCD), density of states (DOS), frontier molecular orbital and recovery property were calculated. It can be observed that the structures of the SO_2_, SOF_2_ and SO_2_F_2_ molecules changed significantly after being adsorbed. Meanwhile, the E_ad_ and Q_T_ of these three adsorption systems are relatively large, while that of the HF adsorption system is the opposite. These phenomena indicate that Au-InN monolayer has strong adsorption capacity for SO_2_, SOF_2_ and SO_2_F_2_, and the adsorption can be identified as chemisorption. In addition, through the analysis of frontier molecular orbital, it is found that the conductivity of Au-InN changed significantly after adsorbing SO_2_, SOF_2_ and SO_2_F_2_. Combined with the analysis of the recovery properties, since the recovery time of SO_2_ and SO_2_F_2_ removal from Au-InN monolayer is still very long at 418 K, Au-InN is more suitable as a scavenger for these two gases rather than as a gas sensor. Since the recovery time of the SOF_2_ adsorption system is short at 418 K, and the conductivity of the system before and after adsorption changes significantly, Au-InN is an ideal SOF_2_ gas-sensing material. These results show that Au-InN has broad application prospects as an SO_2_, SOF_2_ and SO_2_F_2_ scavenger and as a resistive SOF_2_ sensor, which is of extraordinary meaning to ensure the safe operation of power systems. Our calculations can offer a theoretical basis for further exploration of gas adsorbent and resistive sensors prepared by Au-InN.

## 1. Introduction

Nowadays, SF_6_ is extensively applied to gas-insulated switchgears (GIS) because of its excellent insulation and arc extinguishing properties [1,2,3,4]. In cases where the high-voltage insulation equipment has been working for a long time, partial overheating and discharge is very likely to occur, leading to the decomposition of SF_6_. After the decomposition of SF_6_, gases such as HF, SO_2_, SOF_2_ and SO_2_F_2_ will be generated [5,6,7], which will cause aging and even damage to high-voltage insulation equipment [8,9,10]. Previous reports have shown that it is possible to judge whether electrical equipment is malfunctioning through detecting the type and content of the SF_6_ decomposed products in GIS. Thus, finding an excellent gas sensor and adsorbent for monitoring and adsorbing the SF_6_ decomposed products in GIS is of great significance to ensure the normal operation of power systems.

Two-dimensional (2D) materials have been broadly applied in electronics, field-effect devices, supercapacitors, sensing materials, energy storage and other fields [11,12,13,14,15]. Graphene is a typical representative of 2D materials, but in some cases, the zero band gap property of it will restrict its application [16,17]. Thus, researchers are exploring other materials with graphenelike structures and appropriate band gaps. Among these novel graphenelike materials, AlN and InN nanosheets, which belong to group III-V nitrides, have drawn extensive attention due to their excellent semiconducting property [18,19,20,21]. In particular, the high carrier mobility and large specific surface area of InN mean that it has great potential to be applied in the field of gas adsorption and sensing [22,23,24]. A large number of reports have demonstrated that transition metal (TM) modified semiconductor materials have superior sensing performance and strong gas adsorption ability [25,26,27]. Guo [28] et al. found that TM (Pd, Pt, Ag, Au) modified InN was a promising candidate material for detecting NO_2_ gas. Wang [29] et al. found that TM (Ag, Au) doped MoS_2_ was considered to be a promising H_2_O molecule adsorbent. Therefore, the doping of Au atom may enhance the adsorption capacity of InN to SF_6_ decomposed products. Au is a precious metal, but so far few studies have analyzed the effects of precious metal doping on the adsorption ability of InN toward SF_6_ decomposed species.

The adsorption and electronic behaviors of Au modified InN monolayer toward four SF_6_ decomposed products have been studied based on first-principles theory in this work. The E_ad_ (adsorption energy), Q_T_ (charge transfer), DCD (deformation charge density), DOS (density of states), frontier molecular orbital and recovery property were calculated so as to obtain the adsorption properties and sensing mechanism of the Au-InN monolayer for four SF_6_ decomposed products. The results suggest that Au-InN has strong adsorption capacity for three SF_6_ decomposed species except HF. Combining the frontier molecular orbital and recovery property analysis can lead to the conclusion that the gas adsorbent and resistive sensor prepared by Au-InN has great potential for adsorbing SO_2_, SO_2_F_2_ and sensing SOF_2_.

## 2. Computation Methods

All the theoretical calculations based on density functional theory (DFT) were carried out in Dmol^3^ package in this study [30]. To handle the electron exchange-correlation terms, the Perdew–Burke–Ernzerhof (PBE) function within generalized gradient approximation (GGA) method was chosen [31]. The DFT-D method was employed for the further understanding of van der Waals forces and long-range interactions [32,33]. Besides, double numerical polarization (DNP) was adopted for calculation while the DFT semicore pseudopotential (DSSP) method was applied to handle core electron relativity effects [34]. In terms of the setup of Monkhorst–Pack k-point mesh, 5 × 5 × 1 was set for geometric optimization and 10 × 10 × 1 for the calculation of static electronic structure [35]. The energy tolerance accuracy, maximum force and displacement were severally set as 10^−5^ Ha, 0.002 Ha/Å, and 0.005 Å [36,37].

A 4 × 4 InN supercell with 16 In atoms and 16 N atoms was established. In order to avoid adjacent supercell interaction, the vacuum region of InN supercell was set as 15 Å [38]. The lattice constant of the fully optimized InN monolayer is calculated to be 3.62 Å, which is consistent with a previous report (3.63 Å [39]).

Through calculating the value of E_ad_, the interaction strength between substrate material and gas molecules can be roughly obtained. Thus, the most stable adsorption configuration can be found by comparing the value of E_ad_. The calculation formula of E_ad_ is as follows [40]:

(1) $$E_{\mathrm{ad}}=E_{\mathrm{Au}-\mathrm{InN}/\mathrm{gas}}-E_{\mathrm{Au}-\mathrm{InN}}-E_{\mathrm{gas}}$$

In the above formula, $E_{\mathrm{Au}-\mathrm{InN}/\mathrm{gas}}$ denotes the energy of the gas adsorption system, while $E_{\mathrm{Au}-\mathrm{InN}}$ and $E_{\mathrm{gas}}$ denote the energies of Au-InN monolayer and isolated gas molecule, respectively. Besides, the charge transfer (Q_T_) during the doping and adsorption process is analyzed through the Hirshfeld method [41]. A positive value of Q_T_ implies that the analyte acts as an electron donator, and conversely implies that the analyte acts as an electron acceptor [42].

## 3. Results and Discussion

### 3.1. Isolated HF, SO_2_, SOF_2_, SO_2_F_2_ Molecules and Au-InN Monolayer

**Figure 1:**
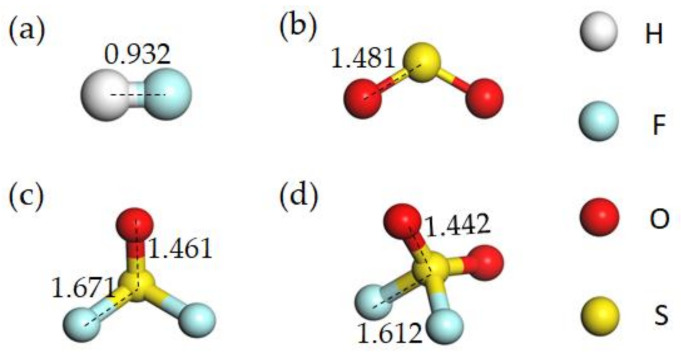
Optimized geometrical structures of (**a**) HF, (**b**) SO_2_, (**c**) SOF_2_ and (**d**) SO_2_F_2_.

The geometrical configurations of four optimized SF_6_ decomposed products—HF, SO_2_, SOF_2_ and SO_2_F_2_—are shown in Figure 1. Meanwhile, the geometrical parameters of four optimized gas configurations are displayed in Table 1, which are basically consistent with previous reports [36,43,44]. Table 2 lists the single atom charges of gas molecules in the gas phase.

In order to obtain the most stable doping configuration for subsequent analysis, four possible doping sites of Au atom are considered [28]. The Au atom could be doped not only directly above the N atom (T_N_) or In atom (T_In_) in the InN monolayer but also right above the In-N bond (T_B_) or at the hollow center of the six-membered ring of the InN monolayer (T_H_). Afterwards, so as to measure the stability of each optimized doping system, the binding energy (E_b_) was calculated. The E_b_ of each doping system is calculated as follows:

(2) $$E_{b}=E_{\mathrm{Au}-\mathrm{InN}}-E_{\mathrm{InN}}-E_{\mathrm{Au}}$$

**Figure 2:**
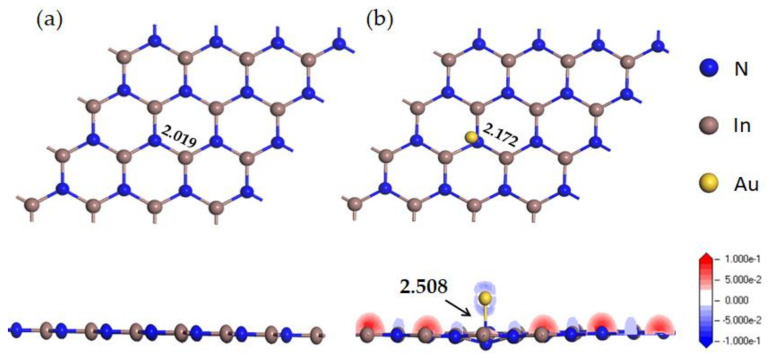
(**a**) The top and side view of InN monolayer; (**b**) The top view of Au-InN monolayer and DCD in side view. Bond length is shown in black.

In the above formula, $E_{\mathrm{Au}-\mathrm{InN}}$ represents the energy of the Au-InN monolayer, while $E_{\mathrm{InN}}$ and $E_{\mathrm{Au}}$ represent the energies of the pure InN monolayer and Au atom, respectively. The negative E_b_ values of the four doping systems in this study indicate that they all emit heat during their establishment. The doping system is the most stable when the Au atom is doped to the T_N_ site, as displayed in Figure 2b, because of the absolute value of its E_b_ is the largest (E_b_ = −1.61 eV). In this doping system, the length of the In-N bond increases from 2.091 Å to 2.172 Å, which shows the strong interaction between the InN monolayer and Au dopant.

From the DCD shown in Figure 2b, the area where the charge density increases is displayed in red, otherwise it is displayed in blue. In the doping system, the positive charge of the Au atom (0.116 e) implies that Au dopant provides electrons to the InN monolayer. It can be found from DCD that the electron accumulation region is principally confined to In atoms while the consumption region is principally confined to N atoms. The relatively large charge transfer and structural deformation suggest the strong interaction between Au dopant and InN monolayer; in other words, the doping structure is very stable.

**Figure 3:**
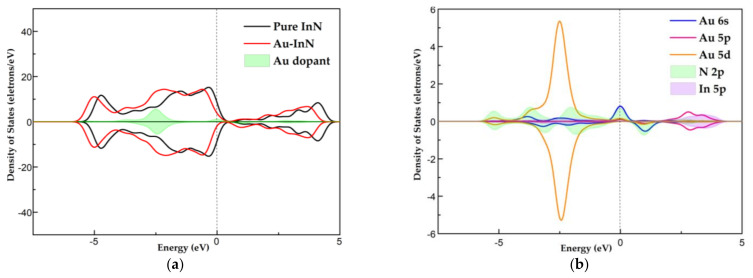
(**a**) TDOS of the system before and after doping. (**b**) PDOS of the doped system, the dotted line indicates the Fermi energy.

Meanwhile, DOS was considered so as to have a better understanding about the electronic behavior of the doping system. As can be observed from Figure 3a, the total DOS (TDOS) of the doping system shifts to the left compared with that of the pure InN monolayer. In addition, the spin up and spin down curves of TDOS in InN are highly symmetrical while those of Au-InN are asymmetric, which suggests that Au doping makes the InN monolayer change from non-magnetic to magnetic [45]. Meanwhile, the TDOS of the entire system shifts to the left, and a new peak appears near −2.5 eV, which means that several impurity states introduced by the doping of Au have changed the electronic behavior of the entire system. In atomic DOS (PDOS) (Figure 3b), it can be observed that N 2p orbital and Au 6s, 5p, 5d orbitals have a considerable overlap near −5.2, −3.5 and 0 eV in the spin up and −5.1, −3.0 and 1.0 eV in the spin down. Besides, the In 5p orbital and Au 6s orbitals overlap near −2.5 eV in the spin up and −2.2 eV in the spin down. Meanwhile, the In 5p orbital and Au 5p orbitals have an obvious overlap near 2.7 and 3.5 eV. These phenomena suggest that the orbital hybridization between the Au atom and the In, N atom is very strong, and a stable Au-N bond is formed. In particular, since the orbital hybridization between Au and N atom is in the vicinity of the Fermi level, the electronic behavior of the entire system will undergo greater changes [46]. In summary, the electronic behavior of the InN monolayer will be significantly changed by Au doping.

### 3.2. Adsorption Behavior of HF, SO_2_, SOF_2_ and SO_2_F_2_ on Au-InN Monolayer

**Figure 4:**
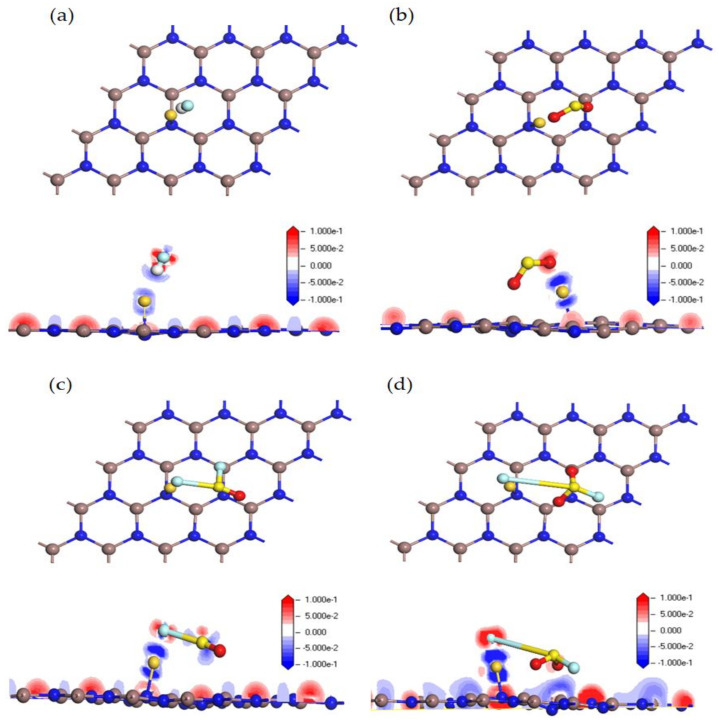
The steadiest adsorption configuration of gas on Au-InN monolayer and the DCD of this configuration: (**a**) HF, (**b**) SO_2_, (**c**) SOF_2_ and (**d**) SO_2_F_2_ adsorption system. The length of Au-N bond is shown in black.

In this section, the adsorption behaviors of Au-InN to HF, SO_2_, SOF_2_ and SO_2_F_2_ are analyzed. The gas molecules are placed on the surface of the Au-InN monolayer in different directions so as to find the most stable adsorption structure for subsequent analysis. Figure 4 displays the steadiest structure of each gas adsorption system, the adsorption characteristic parameters of which are listed in Table 3.

As can be seen from Figure 4a, the HF molecule prefers to be adsorbed at a position vertical to the InN plane with a small slope and the H-F bond elongates from 0.932 Å to 0.957 Å. The slight structural deformation implies the weak interaction between HF and Au-InN. Meanwhile, the small absolute values of Q_T_ (0.05 e) and E_ad_ (−0.31 eV) also prove that the adsorption of HF upon Au-InN is not stable. Thus, Au-InN is unsuitable for detecting and removing HF. In the SO_2_ adsorption system, the most stable configuration bears a resemblance to the HF absorption configuration. After the adsorption of SO_2_, the length of S–O bond increases from 1.481 Å to 1.603 Å. The significant structure deformation indicates that SO_2_ molecule is activated during the interaction with Au-InN [47]. From the molecular point of view, SO_2_ has a negative charge of 0.24 e, indicating the electron-receiving property of SO_2_. Meanwhile, according to the DCD in Figure 4b, the electron accumulation region is principally confined to the S atom. The absolute value of E_ad_ (−1.38 eV) in Au-InN/SO_2_ system is higher than 0.8 eV, hence this adsorption process can be regarded as chemisorption [48]. SOF_2_ and SO_2_F_2_ molecules tend to be adsorbed on the side of the Au dopant in the Au-InN monolayer rather than on the top. In SOF_2_ and SO_2_F_2_ adsorption systems, the S-F bond of SOF_2_ elongates from 1.671 Å to 2.698 Å, while that of SO_2_F_2_ increases from 1.612 Å to 4.776 Å. The more significant deformation of SO_2_F_2_ molecule is related to the larger absolute values of E_ad_ (−2.48 eV) and Q_T_ (−1.12 e) in the SO_2_F_2_ adsorption system. As can be observed from DCD in Figure 4c,d, the electron density around the Au dopant decreases while that around the F atom increases. As electron acceptors, the SOF_2_ and SO_2_F_2_ molecules obtain 0.57 e and 1.12 e from the Au-InN monolayer, respectively. Besides, the relatively large absolute values of Q_T_ in the SO_2_, SOF_2_ and SO_2_F_2_ adsorption systems imply that after the adsorption of SO_2_, SOF_2_ and SO_2_F_2_, the redistribution of the electrons in the entire system causes the electronic behavior of the Au-InN monolayer to be changed. In conclusion, the Au-InN monolayer has large adsorption capacity toward three SF_6_ decomposed species except HF, and these adsorption processes can be regarded as chemisorption. The relatively large absolute values of Q_T_ and E_ad_ in SO_2_, SOF_2_ and SO_2_F_2_ adsorption systems not only reflects the strong orbital hybridization between atoms in excited gas molecules and Au atoms but also shows the good stability of the adsorption structure [49,50].

Table 4 lists the Q_T_ and E_ad_ of Cu-InN/gas and Au-InN/gas systems (the Q_T_ and E_ad_ of Cu-InN/gas system are obtained from previous report [14]). By comparison, it can be found that the absolute value of E_ad_ in Au-InN/gas system is significantly larger than that in Cu-InN/gas system, except for the SO_2_ adsorption system. This result shows that Au atom doping can improve the adsorption performance of InN to HF, SOF_2_ and SO_2_F_2_ more significantly than Cu atom doping. In summary, the Au-InN monolayer has good potential as an efficient SF_6_ decomposed product adsorbent.

### 3.3. DOS Analysis of HF, SO_2_, SOF_2_ and SO_2_F_2_ Adsorption Systems

**Figure 5:**
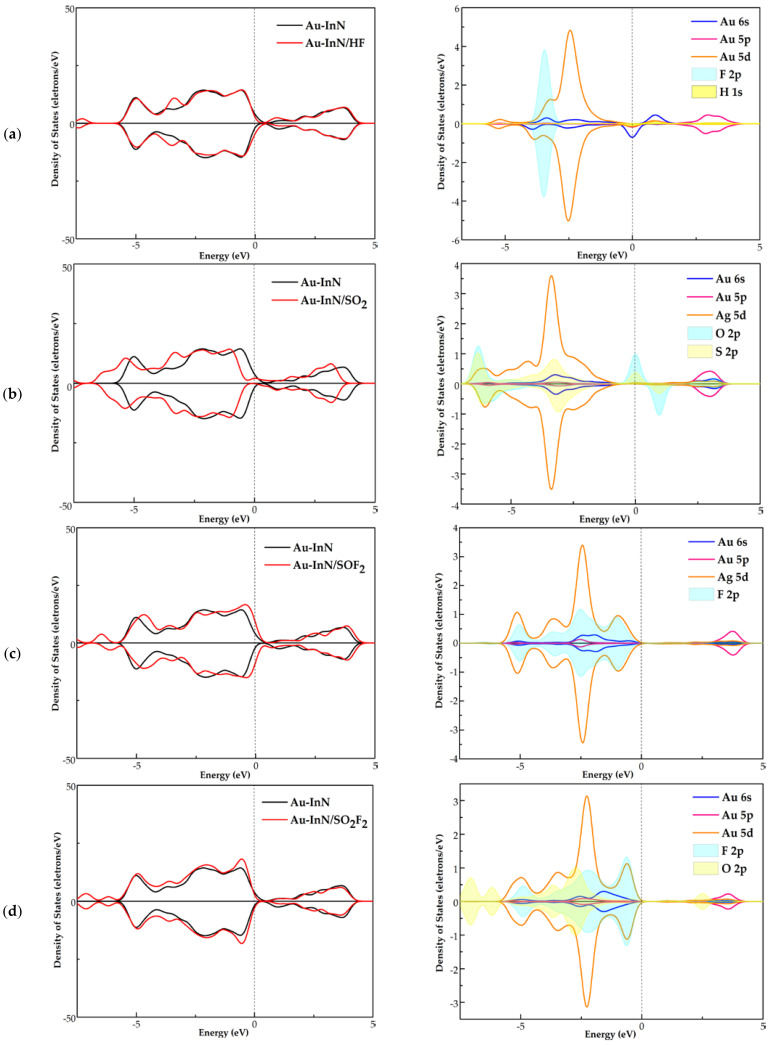
TDOS and PDOS of (**a**) HF, (**b**) SO_2_, (**c**) SOF_2_ and (**d**) SO_2_F_2_ adsorption system, the dotted line indicates the Fermi energy.

Therefore, in order to further study the electron behavior of four adsorption systems, the TDOS and PDOS of the Au-InN/gas systems are investigated. As shown in Figure 5, the TDOS of each adsorption system has varying degrees of deformation in comparison with that of Au-InN monolayer. In the TDOS of HF and SO_2_F_2_ adsorption systems, the gap at the bottom of the guide band in the spin up curve narrowed slightly. This phenomenon implies that the conductivity of the Au-InN monolayer may be changed after adsorbing HF and SO_2_F_2_. The TDOS of the SO_2_ adsorption system has an obvious left shift compared with that of the Au-InN monolayer, and the degree of TDOS deformation is larger than HF, SOF_2_ and SO_2_F_2_ adsorption systems. Besides, since gas molecules are activated during the interaction with the Au-InN monolayer, some novel states appear in TDOS. The spin up and spin down curves of TDOS in HF, SO_2_ and SOF_2_ adsorption systems are asymmetric; in other words, the magnetic property of the Au-InN monolayer does not change after the adsorption of HF, SO_2_ and SOF_2_. However, the TDOS spin up and down of the SO_2_F_2_ adsorption system is highly symmetrical, which implies that the adsorption of SO_2_F_2_ makes the Au-InN monolayer change from magnetic to non-magnetic.

In the PDOS of the HF adsorption system, the hybridization between F 2p orbital and Au 5d, 6s orbitals is in the vicinity of −3.5 eV. In SO_2_ adsorption system, the S 2p and O 2p orbitals of activated SO_2_ have certain hybridization with Au 5d orbital at −6.2, −3.5 eV in the spin up and at −6.0, −3.2 eV in the spin down. As can be seen from the PDOS of SOF_2_ adsorption system, the large overlap area between the F 2p orbital and Au 6s, 5p and 5d orbitals near −5.1, −3.5, −2.5 and −1.0eV indicates the strong interaction between F and Au atom. It can be observed from the PDOS of the SO_2_F_2_ system that the F 2p orbital overlaps with the Au 5d orbital near −5.0, −2.3, −0.5 eV, and the overlap area is bigger than that of the HF, SO_2_ and SOF_2_ adsorption systems. These phenomena show that the interaction between SO_2_F_2_ and Au-InN is the strongest, which supports the large absolute values of E_ad_ and Q_T_ in SO_2_F_2_ adsorption system. Besides, the strong orbital hybridization between the atoms in excited SO_2_, SOF_2_, SO_2_F_2_ and Au atoms verifies the previous conclusion that three SF_6_ decomposed species (except HF) can be stably adsorbed by Au-InN.

### 3.4. Frontier Molecular Orbital Analysis

**Figure 6:**
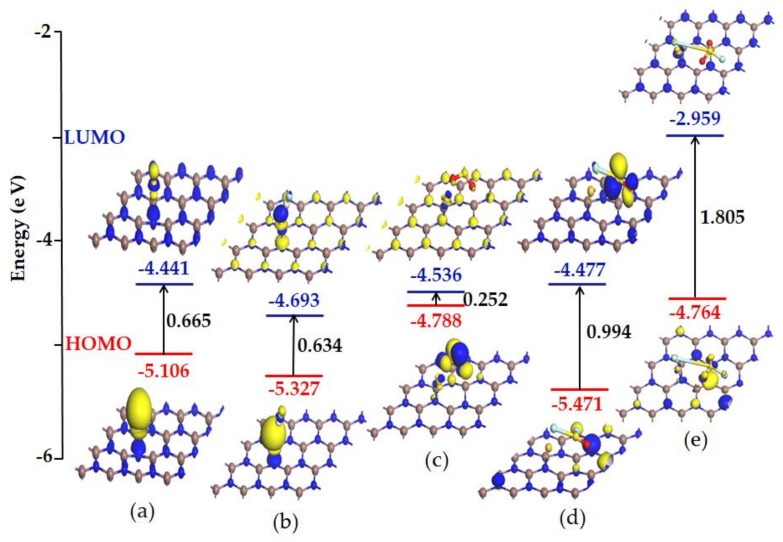
HOMO and LUMO of (**a**) Au-InN monolayer and (**b**) Au-InN/HF, (**c**) Au-InN/SO_2_, (**d**) Au-InN/SOF_2_, (**e**) Au-InN/SO_2_F_2_ systems.

From frontier molecular orbital (FMO) theory, we know that the energy between the lowest unoccupied molecular orbital (LUMO) and the highest occupied molecular orbital (HOMO) is called the energy gap (E_g_). It is a feasible method to measure the conductivity of a material through calculating E_g_. In this section, the effect of gas adsorption on the conductivity of the whole system is investigated. Through analyzing the frontier molecular orbital, we can explore the feasibility of Au-InN as a gas resistive sensor for detecting SF_6_ decomposed species. As can be seen from Figure 6, HOMO and LUMO are mainly located near to an Au atom before gas adsorption, indicating that the Au dopant has strong electron mobility. In the Au-InN doping system, the energy of HOMO is −5.106 eV while that of LUMO is −4.441 eV, and the E_g_ is 0.665 eV. Besides, the E_g_ of the HF, SO_2_, SOF_2_ and SO_2_F_2_ adsorption systems are 0.634 eV, 0.252 eV, 0.994 eV and 1.805 eV, respectively. The obvious change of E_g_ after adsorbing SO_2_, SOF_2_ and SO_2_F_2_ suggesting the adsorption of these three gases has a significant effect on the conductivity of Au-InN. However, in the HF adsorption system, the E_g_ is basically the same as that of the Au-InN monolayer, which shows that the adsorption of HF basically does not affect the conductivity of Au-InN. Therefore, Au-InN may be used as a resistive gas sensor for detecting SO_2_, SOF_2_ and SO_2_F_2_, which is of extraordinary importance to ensure the normal working state of high-voltage insulation equipment.

### 3.5. Recovery Property of Au-InN Monolayer upon HF, SO_2_, SOF_2_ and SO_2_F_2_

The gas sensor must pay attention to the problem of recycling, that is to say, the desorption process of gas molecules from the sensor surface must be considered. Only after a comprehensive analysis of the gas adsorption and desorption processes can the application feasibility of the Au-InN monolayer as adsorbent and gas sensor be further explored. Recovery time is an important parameter for evaluating the desorption capacity of gas-sensitive materials, and its calculation formula is as follows [51]:

(3) $$\tau =A^{-1}e^{\left(-E_{a}/K_{B}T\right)}$$

**Figure 7:**
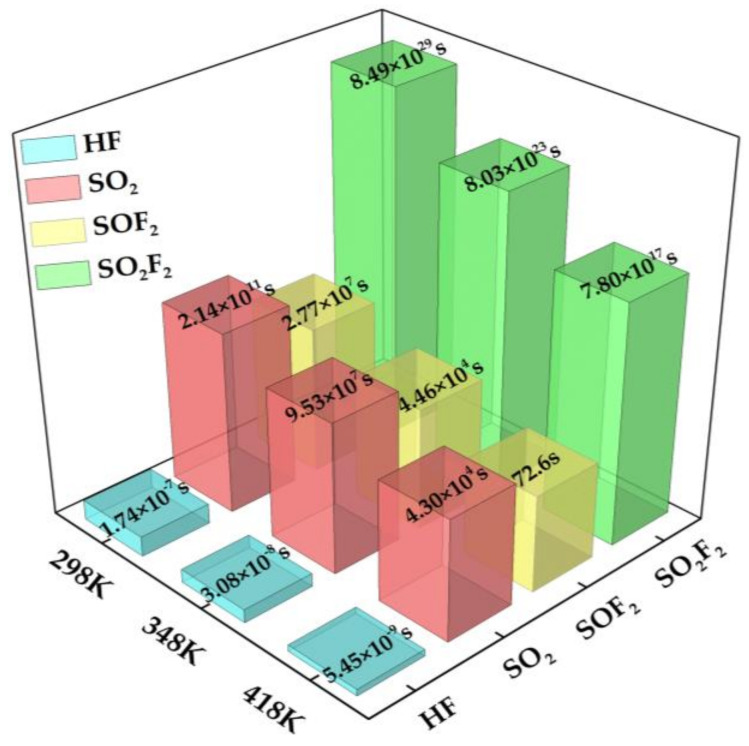
Recovery time of four adsorption systems at various temperatures.

In the above formula, A represents the attempt frequency ($10^{12}\;s^{-1}\;$ [52]), while T and $K_{B}$ are the tested temperature and Boltzmann constant ($8.62\times 10^{-5}\;\mathrm{eV}/K$), respectively. E_a_ represents the potential barrier of the desorption process. Since adsorption and desorption are inverse processes of each other, the value of E_a_ can be equal to that of E_ad_. In order to fully understand the desorption performance of the four SF_6_ decomposition products on the Au-InN monolayer, we used three temperatures of 298 K (ambient temperature), 348 K, and 418 K as test temperatures. The recovery time of four adsorption systems at various temperatures was calculated, as shown in Figure 7. As can be seen from Figure 7, the instantaneous recovery time at 298 K (ambient temperature) indicates that HF is easily desorbed from the surface of the Au-InN monolayer. This phenomenon also confirms the previous conclusion that the Au-InN monolayer has a weak adsorption capacity for HF. In summary, Au-InN is not suitable as an adsorbent and sensor for HF gas. At the same time, it is very difficult to desorb SO_2_ and SO_2_F_2_ from Au-InN at 298 K (ambient temperature), which also confirms the strong interaction between the Au-InN monolayer and these two gases. Although the recovery time is significantly shortened with the increase in temperature, the recovery time of the two adsorption systems is still very long even at 418 K, especially in the SO_2_F_2_ adsorption system. Therefore, Au-InN is very suitable as a scavenger for SO_2_ and SO_2_F_2_ gas in GIS, and thus has great application prospects in the field of ensuring the safe operation of power systems. In addition, SOF_2_ is difficult to desorb from the surface of the Au-InN monolayer at ambient temperature, but the recovery time at 418 K is significantly shorter, about 72.6 s. This result shows that Au-InN has a strong adsorption performance for SOF_2_ at ambient temperature, and can be recycled due to its short recovery time at high temperature, indicating that Au-InN is an ideal SOF_2_ gas sensing material.

## 4. Conclusions

In this study, the most stable doping structure and the adsorption properties andsensing mechanism of four SF_6_ decomposed species (HF, SO_2_, SOF_2_ and SO_2_F_2_) on the Au-InN monolayer were analyzed based on first-principles theory. The main conclusions of this study are listed as below:

1. When the Au atom is doped at the T_N_ site in InN, the doping system is the most stable.
2. The Au-InN monolayer has strong adsorption capacity toward three SF_6_ decomposed species except HF, and the adsorption can be identified as chemisorption. These results indicate that Au-InN can be a promising scavenger for SO_2_, SOF_2_ and SO_2_F_2_.
3. Compared with Cu atom doping, Au atom doping can improve the adsorption capacity of InN to HF, SOF_2_ and SO_2_F_2_ more significantly.
4. Combined with the analysis of DOS, the strong orbital hybridization between the atoms of excited SO_2_, SOF_2_, SO_2_F_2_ and the Au atom can be observed, which not only makes the adsorption configuration more stable but also reflects the good electron mobility of the Au dopant.
5. It can be obtained from the analysis of the frontier molecular orbital and recovery properties that Au-InN has broad application prospects as SO_2_, SOF_2_ and SO_2_F_2_ scavenger and resistive SOF_2_ sensors, which is of extraordinary importance to ensure the safe operation of power systems.

## Figures and Tables

**Table 1:** Geometrical parameters of HF, SO_2_, SOF_2_, and SO_2_F_2_.

Gas	Bond Length(Å)		Bond Angle (°)	
HF	H-F	0.932	-	-
SO_2_	S-O	1.481	O-S-O	119.936
SOF_2_	S-O	1.461	O-S-O	107.190
	S-F	1.671	F-S-F	93.238
SO_2_F_2_	S-O S-F	1.442 1.612	O-S-O	126.682
			F-S-F	94.408
			O-S-F	107.836

**Table 2:** Single atomic charges of gas molecules.

Gas	H	F	S	O
HF	0.345	−0.345	-	-
SO_2_	-	-	0.455	−0.227
SOF_2_	-	−0.251	0.710	−0.208
SO_2_F_2_	-	−0.214	0.870	−0.220

**Table 3:** The characteristic parameters of HF, SO_2_, SOF_2_ and SO_2_F_2_ adsorption systems.

System	The Length of Bond (Å)		Adsorption Distance(Å)	Atom	Charge(e)
Au-InN+HF	H-F	0.957	2.307	H	0.332
	Au-N	2.370		F	−0.379
Au-InN+SO_2_	S-O	1.603	2.093	S	0.352
	Au-N	2.070		O_1_	−0.434
				O_2_	−0.406
Au-InN+SOF_2_	S-F	2.698	2.016	S	0.565
				O	−0.303
	Au-N	2.081		F_1_	−0.294
				F_2_	−0.537
Au-InN+SO_2_F_2_	S-F	4.776	1.984	S	0.593
				O_1_	−0.384
	Au-N	2.087		O_2_	−0.380
				F_1_	−0.532
				F_2_	−0.412

**Table 4:** The charge transfer (Q_T_) and adsorption energy (E_ad_) of Cu-InN/gas and Au-InN/gas systems.

Adsorption System	Q_T_ (e)	E_ad_(eV)
Cu-InN/HF	−0.14	−0.09
Cu-InN/SO_2_	−0.24	−1.85
Cu-InN/SOF_2_	−0.10	−0.85
Cu-InN/SO_2_F_2_	−0.55	−1.02
Au-InN/HF	−0.05	−0.31
Au-InN/SO_2_	−0.49	−1.38
Au-InN/SOF_2_	−0.57	−1.15
Au-InN/SO_2_F_2_	−1.12	−2.48

## Data Availability

The data is available on the request from corresponding author.
